# Bridging Faith and Health: The Role of Muslim Religious Leaders in Disseminating COVID-19 Information in Norway

**DOI:** 10.1007/s10943-025-02368-6

**Published:** 2025-06-30

**Authors:** Seila Mahic, Lise-Merete Alpers, Kari Storstein Haug

**Affiliations:** 1 Master of Nursing–Clinical Research and Professional Development, VID Specialized University, Postbox 184, 0319 Oslo, Norway; 2 Faculty of Health Sciences, VID Specialized University, Postbox 184, 0319 Oslo, Norway; 3 Faculty of Theology, Diaconia and Leadership Studies, VID Specialized University, 4024 Stavanger, Norway

**Keywords:** COVID-19, Health crisis, Information, Religion, Religious leaders

## Abstract

The aim of this study was to explore how Muslim religious leaders in Norway handled COVID-19 information dissemination. A total of six religious leaders were interviewed from two Norwegian municipalities. Interviews were analyzed using the Graneheim and Lundman content method. The religious leaders played crucial roles in disseminating information, using religious justifications to reinforce public health messages and combat misinformation. Trust between religious leaders and authorities is vital for effective collaboration. Religious leaders should be included in emergency plans as bridges between authorities and communities. Insights from this study suggest including religious leaders in emergency plans during health crises.

## Introduction

During the COVID-19 pandemic, tens of millions of people were infected worldwide, leading to over 7 million deaths (World Health Organization [WHO], 2024). However, the pandemic also had consequences beyond the medical victims of the virus. When the WHO declared the virus outbreak a pandemic on 11 March 2020 (WHO, 2020b), several countries implemented restrictions to slow the spread of the virus, including lockdowns in 82 countries (UNICEF, 2020). In addition to encouraging social distancing, the WHO (WHO, 2020a) called attention to the importance of being well informed about the disease and how the virus spreads, as there were no vaccines or curative treatments at the beginning of the pandemic.

As the pandemic unfolded, some immigrant groups were overrepresented in infection and hospitalization rates in many parts of the world, including Norway (NOU, 2022; OECD, 2022). Several possible reasons have been presented to explain this, including socioeconomic conditions, lack of infection-prevention behaviors, low health literacy, social contact within groups, international travel, lack of digital competence, and language barriers (Brekke et al., 2022; Indseth et al., 2020; Nordic Council of Ministers, 2022). The immigrants with the highest incidence of infection in Norway were from Somalia, Pakistan, Iraq, Turkey, and Morocco (FHI, 2021). When vaccination became a possibility, immigrants were also underrepresented among those who got vaccinated (OECD, 2022).

The authorities in Norway initially struggled to disseminate COVID-19 information to the immigrant population (Barstad, 2021; NOU, 2022), primarily due to insufficient information and failed communication (Kjeøy et al., 2023). To reach all parts of the immigrant population, the Norwegian authorities recognized the importance of involving resource persons, including religious leaders from immigrant communities.

During life-threatening situations or in times of crises, research has shown that people may turn to religion for comfort and explanation, using it as a coping strategy to reduce their insecurity (Bentzen, 2021; Molteni et al., 2021). Religious meetings and environments are meaningful for believers, and they can be even more important for some immigrants (Sisti et al., 2023). Immigration often leads to separation from family, relatives, and friends. Therefore, the opportunity provided by religious fellowships and congregations to meet and cultivate social relationships with other believers and people from similar ethnic backgrounds is essential (Buonsenso et al., 2020). Furthermore, religion in general is important for many immigrants, as it helps them protect and maintain their cultural background (Jacobsen et al., 2018).

One of the most important religious health assets may be religious leaders (Tan et al., 2022). Throughout history, religious leaders have played a key role in health crises by communicating helpful information and promoting health-saving practices, helping to prevent and reducing fear and stigma, and providing reassurance to people in their communities (WHO, 2021). In addition, religious leaders are typically skilled in public communication and can thus influence thinking and shape social values and social responsibility through their preaching (Tan et al., 2022).

Following the restrictions that required social distancing, many religious leaders and communities paused or adapted interpersonal activities and limited public events to help curb the spread of COVID-19. While some religious leaders opposed COVID-19 information and restrictions from authorities, many contributed to help prevent infection, using their religious authority to support the measures implemented to control the virus outbreak (Borchgrevink et al., 2020).

Authority is a central part of many religions. Bøe and Farstad (2023) highlighted that authority exists in different forms and can be expressed in different ways. When considering authority in relation to Islam, it is relevant to talk about a mix of legal and traditional authority (Bøe & Farstad, 2023, p. 41). Imams, for example, define their authority by pointing to tradition, knowledge, and the opportunity they have to reach out to many people (Døving, 2012, p. 39). Furthermore, in Islam, there is a certain type of knowledge that bestows the right to interpret sacred sources, and this is what forms the basis of a leader’s authority. Importantly, it is the scholars (*ulama*) and not the imams who initially possess this authority (Døving, 2012, p. 27). Therefore, it is not a given that an imam will be a scholar and possess the legitimate power to interpret sacred sources. In addition, Muslims adherence to and trust in religious leaders is crucial for their authority (Bøe & Farstad, 2023).

Some studies have explored the involvement and impact of religious leaders during the COVID-19 pandemic. A study on religious leaders in rural Christian, Catholic, Jewish, and Muslim communities in the USA showed that they used faith and religious tenets as foundational principles to encourage compliance with health recommendations (Hopgood et al., 2024). Another US study of religious leaders in a tight-knit Modern Orthodox Jewish community demonstrated their crucial role in promoting the well-being of their members. They accomplished this by organizing community outreach, mobilizing tangible services, conducting virtual religious services, and relaying health-related information (Weinberger-Litman et al., 2020). Research from Norway has also highlighted how Islamic religious leaders took responsibility for their members by arranging digital meetings that allowed the public to pose questions about COVID-19 to both imams and medical doctors (Mahic et al., 2023). In addition, healthcare chaplains played a crucial role during the pandemic by addressing the increased social isolation and mental health concerns among patients, families, and healthcare staff. They adapted by developing new and creative methods to provide spiritual care, thereby helping to meet the heightened psychosocial and spiritual needs of those affected (Kwak et al., 2021).

Although some previous studies have explored the involvement of religion and religious leaders during the COVID-19 pandemic, including their role in information dissemination, there is little knowledge regarding religious leaders’ own experiences in reaching out with adapted COVID-19 information, particularly to immigrant populations. Given that the majority of the immigrants in Norway who were overrepresented in terms of infection and hospitalization rates during the pandemic were of Muslim background, the purpose of this study was to investigate the experiences and assessments of Muslim religious leaders in Norway regarding their COVID-19 information work. The study also sought to explore their cooperation with authorities and propose measures for improving information-related practices in the future health crisis.

## Methods

### Design

In this study, we used a qualitative, interpretive, and explorative design based on the hermeneutic methodology and followed the Consolidated Criteria for Reporting Qualitative Research (COREQ) (Tong et al., 2007). An interpretive approach captures themes and patterns within subjective perceptions and generates an interpretive description (Thorne et al., 2004). Meanwhile, an exploratory approach is relevant when knowledge about a topic is limited and new angles and information of the topic are desired (Kvale & Brinkmann, 2015). Gadamer’s philosophical hermeneutics focuses on how interpretation and dialog with the text can lead to a new understanding of the phenomenon under investigation (Gadamer, 2012). The text in this study consisted of transcribed interviews.

### Participants

Participants for the study were recruited through purposive and snowball sampling. Purposive sampling involves selecting participants who are “information-rich” and possess relevant experiences regarding the phenomenon being studied (Malterud, 2017; Patton, 2014). Islamic religious leaders, such as imams, daily managers of mosques, and other key resource persons within the mosques, who played an active role during the COVID-19 pandemic by informing and guiding people about the viral disease, were included (see Table 1). The first author (SM) and the second author (LMA) recruited participants by visiting various mosques in Oslo and handing out written requests. Additionally, the first author (SM) sent several e-mail requests to prospective participants recommended by other participants and to individuals identified as relevant through online searches. In the written request, all participants were offered an interpreter. However, they all declined, indicating that they had no need for one since they spoke and understood Norwegian fluently. After six interviews, we considered data saturation to have been achieved, as no new themes emerged from the participants’ experiences, and additional data only served to confirm our understanding (Flanagan & Beck, 2024). The religious leaders were recruited from two different cities in Norway.

**Table 1:** Participant Demographic Characteristics (n = 6)

Religious leader	Gender	Title/ Role
1	M	Imam
2	M	COVID-19 Coordinator and Board Member in a Mosque
3	M	Imam
4	M	Daily Manager of a Mosque
5	M	Imam
6	M	Board Member in a Mosque and Muslim Organization

### In-depth Interviews: Digital and In-person Interviews

In-depth interviews were utilized for data collection, which occurred between June 2022 and February 2024. Four of the interviews were conducted digitally through Teams at the participants’ request, while two preferred to meet in person. These two interviews were conducted at the participants’ workplace. A semi-structured interview guide was used for the interviews, including specific topics or questions to cover during conversations with each participant (Flanagan & Beck, 2024; Kvale & Brinkmann, 2015, p. 162). In this study, the topics included the role of religious Muslim leaders in relation to the pandemic, their experiences with information work, challenges with this work, and potential measures to improve their practices in the event of a similar crises when their assistance with information is needed. The interviewer motivated participants to discuss all the topics on the agenda openly and to share stories in their own words. This approach ensured that researchers gather all the necessary information while allowing participants the flexibility to offer as many examples and explanations as they desire (Flanagan & Beck, 2024, p. 503). The topics raised in the interviews were not considered sensitive. Therefore, digital interviews were advantageous for the study, as they reduced the participation barrier and allowed relevant participants located far from the researcher to be included (Thunberg & Arnell, 2022). The first author (SM) conducted all the interviews with each participant in Norwegian, recorded them, and transcribed them verbatim. Furthermore, the first author (SM) translated the quotes used in the results section into English. The interviews lasted from 18 to 47 min.

### Data Analysis

Data were analyzed using the inductive content analysis method described by Graneheim and Lundman (2004), including both manifest and latent approaches. The manifest approach indicates what the text says and describes the visible, obvious components, while the latent perspective refers to the interpretation of the underlying meaning of the text (Graneheim & Lundman, 2004). The analysis consisted of five steps: selecting quotes that contain meaning units related to the research aim, condensation of the data to reduce the text into manageable meaning units, labeling the condensed meaning units using codes, organizing the codes into categories, and finally, merging the categories into themes. In inductive content analysis, the heading of a category describes the content on a manifest level with a low degree of interpretation, while a theme is always an interpretation and therefore describes the content on a latent level (Graneheim et al., 2017). The first author (SM) conducted the analysis in collaboration with the other two authors (LMA and KSH). The first and second authors (SM and LMA) are nurses who have worked with immigrants in various settings, as well as conducted research on health and migration. The last author (KSH) is a theologian and has extensive research experience in the fields of religion and migration. All the analytic steps were discussed by the authors to ensure the trustworthiness and credibility of the analysis. The analysis process resulted in five categories and three themes (see Table 2), which are described in more detail in the results section.

**Table 2:** Themes and categories

Themes	Category 1	Category 2	Category 3
The Muslim Leaders’ Leadership during the COVID-19 Pandemic	Communication and Clarification of Information through Various Channels	The Use of Religious Justifications to Reinforce Authorities’ Messages	Clarification of Theological Questions and the Fight against “Fake News” and Rumors
The Muslim Leaders’ Cooperation with the Authorities in Crisis Situations	Law-Abiding Religious Leaders	The Importance of Trust in Collaboration	
The Muslim Leaders’ Suggestions for Improvement in the Event of a New Pandemic or other Crisis	Information in Advance	Consolidation of Cooperation between Muslim Religious Communities and the Authorities	

### Ethical Considerations

The Norwegian Agency for Shared Services in Education and Research (Sikt) assessed and approved the study (Reference Number 106529), and the study was conducted in accordance with the principles of the Declaration of Helsinki (World Medical Association, 2008). All of the participants provided written consent after being informed in writing about the aim of the research, that it was voluntary, and that they could withdraw their consent at any time. All identifying information was removed when working with the findings to ensure anonymity, and all the recordings were deleted after transcription.

## Results

### The Muslim Leaders’ Leadership during the COVID-19 Pandemic

#### Communication and Clarification of Information through Various Channels

All the religious leaders in this study indicated that their primary role during the pandemic was to communicate the significance of adhering to the prevailing restrictions and infection control measures. When the mosques had to be closed and it was not possible to gather due to the restrictions, one of the participants reported using various information channels to reach out with information to their members:

*We used social media actively, and it was mainly Facebook, and then there was the mosques website, and of course we have our members to whom we send text messages.* (RL3)

In this quote, the participant explains that religious leaders varied in the way they shared information about COVID-19. Different information channels were used, as the religious leaders adapted the way they provided information based on the different age-groups in their Muslim networks. For younger members, social media, such as the Facebook pages of the mosques, were important sources of information. In contrast, the oldest members, who were not active on social media and who lacked digital skills, often received information orally through telephone calls. Eventually, some mosques also started to share video transmissions about COVID-19, which were made by doctors from various immigrant communities. In connection with this, one of the participants described how they encouraged younger individuals to assist the older ones in accessing the mosques’ video transmissions:

*When doctors and similar professionals were available to provide information, we actually asked the younger members or close relatives to facilitate for their parents, grandparents or those unable to connect… So, we encouraged the younger generation to assist the older members presenting information online.* (RL6)

Providing information orally was described by some religious leaders as a particularly important measure to reach more elderly immigrants, who were particularly vulnerable to COVID-19. One of the participants justified this as follows:

*We are oral people. We speak, more than we read and write.* (RL3)

This quote illustrates the importance of being familiar with different cultures in the different immigrant communities when presenting important health information.

### The Use of Religious Justifications to Reinforce Authorities’ Messages

Several of the participants said they used theological justifications to make people understand the seriousness of communicating the authorities’ guidelines and infection control rules as effectively as possible to their fellow Muslims. One of the participants expressed this as follows:

*...we used a lot of theology. Since the prophets’ time, over 1400 years ago, there is a Hadith (corpus of the sayings or traditions of the Prophet Muhammad), where he talks about a pandemic and says, “that if there is a pandemic in an area, then people should not go to that area, and people from that area should not leave the pandemic area either”. So, it is some form of isolation, right? And this is from over 1400 years ago, so there was probably a pandemic or something at the time it was then recommended.* (RL2)

This participant pointed out that the Prophet Muhammad’s guidance on pandemics was referenced to clarify the messaging about social distancing and isolation. Several of the religious leaders also indicated that they used a specific Quranic quote to highlight the importance of taking care of oneself and those in vulnerable situations, as in the following extract:

*...it is important to take care of life and health, and that the greatest responsibility in a Muslim is to bear that responsibility for himself, but also for society and those around him, and saving one person is like saving all of humanity. So, it is the classic we have used during the Corona pandemic that: “Saving one person is like saving all off humanity.”* (RL3)

Here, it is emphasized that Muslims have a human responsibility and that the Quranic quote can be interpreted as an encouragement to be helpful and show care.

### Clarification of Theological Questions and the Fight against “Fake News” and Rumors

Some of the religious leaders experienced that some in the Muslim community did not believe that the viral disease was real, and, especially at the start of the pandemic, they faced challenges reaching out to certain immigrant groups within the Muslim community due to:

*Fake news, or rumors, that went on WhatsApp, or that went on other social media, in relation to: “No, there is no such thing as Corona.”* (RL2)

In this quote, the participant notes that rumors, which abounded on social media, were an obstacle in their information work. Examples of rumors mentioned by the participants included the goal of reducing the world population and the idea that women who got vaccinated could not get pregnant. Another type of understanding that also spread, but which had a more theological significance, was that one should not be vaccinated during Ramadan (i.e., while one is fasting). One of the participants said that several Muslim networks came together to clarify various issues, such the vaccine and fasting:

*Then it was the Muslim leading institutions that came to the conclusion that it is permissible to take the vaccine, and that is would not affect your own fasting, because it does not give you nourishment. So, it went well.* (RL3)

Various issues were discussed within Muslim networks, ultimately clarifying information that was in line with the authority’s advice. Several of the religious leaders agreed that such quick explanations are crucial in dealing with rumors in crisis situations. At the same time, one of the participants pointed out that the message does not always get across:

*With some, of course, it will never be useful because, since they have some thoughts in their head, that’s how it is and that’s how it will be, finished. But with other it can be useful to discuss and talk about things. Some of these are willing to perhaps change their minds.* (RL5)

### The Muslim Leaders’ Cooperation with the Authorities in Crisis Situations

#### Law-Abiding Religious Leaders

Several of the religious leaders said that they constantly followed developments related to the disease on the news, and that they were early on discussing in the board of the various mosques, including whether and how they should maintain various activities during the first closure of society. One of the participants stated the following:

*I think it was good that we started discussing it early so that you also start thinking about measures in parallel. The state and municipalities also started that discussion. On March 12, there was a press release about closing the mosque and everything. So did MDN (Muslim Dialogue Network), and they gave notice to cancel all agreements and projects. There was no discussion when we closed. Out members were all prepared, and we did have measures in place. Like we closed the schools (Quran school) in the mosque, and we went straight to the online platform.* (RL4)

Here, the participant describes that those in the mosque were prepared and had established systems to handle various incidents during the pandemic. Thus, they could, for example, accommodate the authority’s shutdown while at the same time safeguarding the religious activities in the mosques by offering them digitally. One of the participants, who was an imam himself, said that they too were constantly told by either the mosque management or the superior imam for the various congregations to follow the authorities’ advice and regulations:

*It was one and the same message all the time, that we must listen to the authorities and that we must be careful. Therefore, we tried to take care of everything that the authorities gave instructions about.* (RL1)

The participant points out how the various religious leaders in the Muslim community constantly encouraged each other to be law-abiding by following instructions from the authorities.

#### The Importance of Trust in Collaboration

Several religious leaders emphasized how important it was for them to have trust in the authorities’ handling of the pandemic. One of the participants described the significance of trust as follows:

*Trust was mostly about trust in the authorities who actually run the country. It is crucial. If everyone starts to doubt the government’s intuitions, then everyone will just go in their own direction, and then there will be chaos. We must have a government where we can all stand behind and work towards the same goal. I think all religious communities and many other actors in civil society have played an important role and collaborated well with the authorities here.* (RL5)

This participant emphasized that several Muslim leaders were loyal to the authorities. Indeed, that was the message they wanted to convey to the members of the various mosques, so that they too would cooperate and support the authorities in their handling of the pandemic. Another participant described the close cooperation between the authorities and the religious networks as extraordinary:

*...I think it must be the first time in Norway’s history where the municipality and all health authorities realized how important faith and belief communities are. They realized we have a very important role. Because they tried to spread the information themselves at first, but they couldn´t do it. Suddenly, the infection rate began to increase in various districts, among different groups with different immigrant backgrounds, and they couldn’t reach them. So, they saw that we could play a role. We thought it was very good to be able to contribute. At the same time, I think Norway stands out greatly among other countries. Where we have the closeness to our politicians and perhaps the trust that doesn´t exist in many other places.* (RL4)

This religious leader expressed pride in being able to represent the Muslim community and having the opportunity to contribute and cooperate with the authorities on information work. He emphasized that Norway stands out positively from other countries in term of mutual trust between people and the authorities. In addition, he pointed out that people in Norway have a certain proximity to the authorities that provides a greater opportunity to be heard. However, some of the religious leaders indicated that they did not receive public recognition for their contributions, and it is something they would appreciate in the future:

*...Some more public recognition for the work is called for. Because it also has to do with the motivation for those who work in this field. There are many individuals involved and it is not always acknowledged, especially for the purpose of building trust and for the youth, it is essential for identity formation and important for the next generation. This fosters a sense of “Okay, our parents made an effort, and it was well received, yes, this is our country” … Therefore, it is incredibly important for this work to continue, and for faith communities to receive public recognition, not just with closed circles, so that is reaches out to the general public to some extent.* (RL2)

In this excerpt, the participant articulates how public recognition of contributions can strengthen the trust between the collaborating parties and provide motivation to engage in community-oriented work. In this way, recognition could encourage more people to contribute to the community and thus enhance the sense of belonging to Norwegian society. Additionally, they can serve as role models for future generations of immigrants.

### The Muslim Leaders’ Suggestions for Improvement in the Event of a New Pandemic or other Crisis

#### Information in Advance

Several of the religious leaders stated that they needed to constantly keep up with the news about the development of COVID-19 in society, the new restrictions, and measures presented by the authorities at press conferences. As one of the participants noted, they wished they could receive direct information from the authorities:

*We would have liked to have received that information a little in advance so that we were prepared when these press conferences came.* (RL2)

This quote illustrated that the cooperation between religious leaders and the authorities during the pandemic was not entirely coordinated. This participant stated that advanced information could have given them more time to familiarize themselves with the implications of the new information presented by the authorities, thereby enhancing their preparedness.

#### Consolidation of Cooperation between Muslim Religious Communities and the Authorities

Muslim religious leaders were quick to close the doors of mosques and played an important role in limiting the spread of the virus by providing information and guidance to their members. They reached out to certain immigrant groups that the authorities did not have channels to reach. Several participants in this study emphasized the importance of the cooperation that arose between Muslim communities and the authorities. As Muslim immigrants themselves, they have unique knowledge about the environments from which they originate from. One of the participants stated the following:

*So, all that cooperation, it was unique. One can take it as a good example for the future. I also believe that the government saw the value, at least, I hope they did - that immigrants often can help each other – because they understand each other, they communicate in a way and language so that they understand each other, they use the same language codes, they have the same linguistic and cultural background….* (RL3)

This participant emphasized the importance of maintaining cooperation beyond the COVID-19 pandemic, as Muslim religious communities have proven to represent an important resource for the authorities. This quote also clarifies the advantage religious leaders have due to their cultural competence and how important it can be to people with a similar background. However, another participant pointed out that the authorities still have work to do in navigating this cooperation in the future:

*I think the authorities still have big job, in a way, to program for this cooperation, so that it is also taken care of in the next crisis preparedness and that it is also included in the emergency plan – whether it´s terrorism, pandemic or another crisis.* (RL2)

In this quote, the participant notes that the role of religious communities should be included in emergency plans during various crisis situations so that authorities will understand how to include religious leaders if needed.

## Discussion

In this study, we explored how Muslim religious leaders in Norway experienced and assessed their COVID-19 information work. In addition, we examined their cooperation with the authorities and discussed their suggestions for improvements in the event of similar health crises.

### The Different Roles Muslim Religious Leaders Played during the Pandemic

According to the literature, the main roles of religious leaders in Norwegian mosques are leading prayer, conveying Islamic messages to believers, and teaching children and adults to read and understand the Quran (Døving, 2012). Our participants indicated that they also took on additional roles during the pandemic, which they felt were part of their religious leadership responsibility. For example, they contributed by providing information, serving as a link to the authorities, playing an important crisis management role, and being involved in preparedness. Other research has also shown that both Muslim and Christian religious leaders acted as powerful bridges between health authorities and communities during the pandemic (Essa-Hadad et al., 2022). Furthermore, our findings indicate that Muslim religious leaders considered it their main duty during the pandemic to pass on information from the authorities about COVID-19 and to convey the importance of complying with restrictions to their community members. Additionally, several of the participants in our study reported that they employed various measures to reach out and provide information to people in their communities, including tailoring their approach for different age groups. This ensured that all members received the right information, regardless of their digital competence and reading and writing skills. Previous research has reported that religious leaders can identify the unique needs of their community and take necessary measures to protect vulnerable members (Essa-Hadad et al., 2022; Hopgood et al., 2024). Religious leaders’ unique knowledge about the environments they represent can thus be seen as an important resource for the municipality and state (Indseth et al., 2021).

### Religious Reasons and Motivations

A central theme running through the participants narratives about their role in informing and conveying important messages regarding COVID-19 was the use of religious messages. Our participants stated that Quran Hadiths (corpus of the sayings or traditions of the Prophet Muhammad), were important for clarifying messages from authorities regarding social distancing and isolation, along with surah 5:32 (chapter: verses) from the Quran, which implies that saving one person is like saving all of the humanity. Likewise, Hassan et al. (2021) found that religious texts were important in justifying the closure of mosques and disruption of religious practices, such as breaking the fast in congregation at the end of Ramadan. In line with our findings, Begović (2020) noted that also other religious communities, including the Serbian Orthodox, Catholic, and Jewish communities in Bosnia and Herzegovina, found support in their holy books, religious laws, and theological views for COVID-19 restrictions. The arguments shared among these communities as well as in the Islamic community in Bosnia and Herzegovina emphasized the value of human life and the virtue of taking care of the community’s well-being, which is consistent with the findings of our research.

### The Authority of Muslim Leaders

Several of our interviewees mentioned that they came together to discuss and clarify various rumors and what they described as “fake news.” They also highlighted that the idea of vaccination while fasting created uncertainty among some Muslim believers in terms of whether it could be seen as a form of sustenance. Our participants reported the value of quick clarification that supported the authority’s advice when dealing with rumors in their communities. Viskupic and Wiltse (2022) conducted a survey experiment in South Dakota, finding that religious leaders had a positive and statistically significant effect on people’s decision to get vaccinated against COVID-19. In contrast, they found that messages from political or medical leaders had no statistically significant effect. Their results strongly suggest that religious leaders are more effective messengers than other potential messengers and that health authorities would be well served to coordinate their efforts with leaders in faith communities (Viskupic & Wiltse, 2022). Nevertheless, as religious leaders, our participants encountered people who were not interested in the COVID-19 information they presented, as these individuals maintained their own beliefs about the pandemic. In this regard, it is interesting to consider how the authority of religious leaders is perceived in today’s society. Døving and Thorbjørnsrud (2012) argued that religious leaders generally do not enjoy the same respect as they did just a few decades ago, and in general, they are no longer regarded as belonging to the cultural, social, political, or economic elite. It can therefore be debated whether notions of religious leaders are based more on collective memories of powerful historical cultural leaders than on actual realities. Nevertheless, religious leaders were a trusted and notable ally in the response to the pandemic, and our findings confirm that their authority was important during this crisis situation. Hence, we agree with Viskupic and Wiltse (2022) that health authorities would benefit from cooperating and coordinating their efforts with leaders in faith communities.

### The Role of Trust in Collaboration with Authorities

Several of the religious leaders in our study emphasized the importance of trust in their cooperation with authorities. Trust is often said to be society’s glue, lubricant, and foundation, and it makes collaboration easier (Grimen, 2009). A Norwegian report illustrated that the pandemic led to a need for knowledge about what trust means for immigrant communities’ responsiveness to authorities (Brekke et al., 2022). Our participants reported that trust played an important role in their cooperation with the authorities, and they showed their trust by being loyal and law-abiding and by passing on these values to the members of the various mosques in Norway. This finding is consistent with prior research. For example, Mahic et al. (2023) highlighted that religion and religious leaders promoted trust in authorities and their handling of the pandemic. This can be explained by the fact that people more readily trust someone who is similar to themselves and has the same world view and values than someone who is different from themselves (Grimen, 2009). Building on this foundation of trust, our participants encouraged healthcare professionals within their faith communities to share their knowledge about COVID-19, thereby integrating essential health information into community activities. By involving trusted healthcare professionals, the leaders were able to reinforce messaging related to COVID-19.

Indeed, the COVID-19 pandemic has taught us that successful information dissemination depends on a mutual relationship in which the population trusts the authorities and does not believe that they will abuse that trust or take advantage of the extraordinary conditions during a health crisis. Grimen (2009) emphasized that creating trust often involves making the long-term benefits of cooperation outweigh the short-term gains of not cooperating. With this in mind, he highlighted some ways to create and maintain trust, such as by changing the roles related to who helps whom and who takes the first step (Grimen, 2009). Our data show that the participants helped authorities reach immigrants who they lacked the resources to reach themselves. However, our findings do not indicate that the participants expected any specific help in return, although they would have appreciated if their contributions and collaboration with the authorities had been made more visible to larger parts of society.

### Public Acknowledgment of Religious Leaders

According to Døving and Thorbjørnsrud (2012), the usefulness of the services religious leaders perform for the population are rarely discussed outside of religious forums, and thus these services are rarely recognized as important contributions to society. Public debates about religious leaders seem to mainly revolve around negative aspects of their activities, and religious leaders in congregations dominated by minority groups may even be accused of preventing their members from integrating into society (Døving & Thorbjørnsrud, 2012). Our findings suggest that positive recognition by the authorities, in the form of public acknowledgment for the contributions of religious leaders, can enhance trust in the authorities and improve the reputation of religious leaders. On the other hand, one could argue that this could be challenging for authorities, who are already under pressure during crisis situations. Nevertheless, Wijesinghe et al. (2022) emphasized the need to highlight the positive stories not brought to the attention of the public in connection with the collaboration between the government and religious communities, arguing that promoting such partnership could be beneficial in numerous countries. Our participants also reported the importance of maintaining cooperation beyond the COVID-19 pandemic, as the collaboration has proven to be valuable and beneficial to the community. However, some participants indicated that the authorities have some work to do in navigating this cooperation in the future. Accordingly, we suggest that authorities develop an emergency plan that includes religious leaders and faith communities, clearly outlining their responsibilities in information dissemination during crises. More organized and closer cooperation could help optimize the role of religious leaders as a bridge between the authorities and communities. This approach could provide religious leaders with better knowledge of the authorities’ next steps in dealing with crises, giving them more time to prepare before they have to pass on the information.

## Limitations

The findings of this study should be considered in light of certain limitations. First, as noted by Malterud (2002), qualitative data are tied to the text from the interviews and limited by the context of the participants and the place where the study was carried out, in this case Norway. Thus, while data saturation was reached with our sample, the transferability of the findings may be limited to the areas in Norway from which the participants were recruited. However, to increase the transferability of our findings, we followed Malterud (2002) suggestion and provided detailed descriptions of the participants’ experiences and discussed all the analytic steps to enhance the trustworthiness and credibility of the findings. Secondly, one might consider that a purposive sampling strategy combined with the snowball method could result in homogeneous material. Malterud (2017, p. 59) asserts that a purposive sampling strategy emphasizing diversity can provide better informational strength and challenge conclusions that may initially seem obvious. To ensure variation in the data, we recruited participants from various mosques, different branches of Islam, and two distinct cities in Norway. In connection with this, we collected and analyzed data until new information only reinforced our existing understanding (Flanagan & Beck, 2024). However, data saturation can be subjective, as what one researcher considers to be data saturation might not meet the criteria for another, complicating definitive claims of having achieved saturation (Fusch & Ness, 2015).

Third, the primary focus of this study was on Muslim religious leaders, but the inclusion of key authorities could have provided richer data, considering their cooperation with religious leaders. However, by only focusing on the experiences of religious leaders in this research, we provide unique depth and “thick descriptions” of the data material (Dalen, 2011; Malterud, 2017).

## Conclusion

Our study provided in-depth knowledge about how Muslim religious leaders experienced and assessed their COVID-19 information work. The results showed that they conveyed and explained information to their communities through various channels, using religious justifications to reinforce the authorities’ messages. Additionally, they actively combatted “fake news” and rumors about the virus that contradicted the prevailing information and guidelines provided by health authorities. The study also revealed that Muslim religious leaders were law-abiding and appeared to be partners that the authorities could trust.

Furthermore, our study illuminated the role of trust in cooperation between religious leaders and the authorities. Public acknowledgment by authorities of the contributions made by religious leaders was found to have a positive effect on the religious leaders’ motivation to engage in the future cooperation. Our findings highlight the value of continuing to build on this collaboration, as religious leaders’ ability to reach out to other immigrants has proven beneficial to the authorities. This underlines the need for municipalities and the state to focus more on religious leaders as a valuable resource and to include them in emergency plans. In conclusion, the insights gained from this study may help authorities worldwide recognize the benefits of facilitating cooperation with religious leaders during future pandemics or other health crises. As our findings offer significant insights into the role of Muslim religious leaders during the COVID-19 pandemic, we suggest that further research should explore the role of religious leaders from other faiths during the pandemic and compare these findings with those related to Muslim leaders.

## Data Availability

Data generated during the present study cannot be shared due to the need to preserve the informants’ privacy and confidentiality.
